# Latexin inhibits the proliferation of CD133+ miapaca-2 pancreatic cancer stem-like cells

**DOI:** 10.1186/1477-7819-12-404

**Published:** 2014-12-30

**Authors:** Zhan-Xiong Xue, Ji-Hang Zheng, Zhi-Qiang Zheng, Jing-Li Cai, Xiao-Hua Ye, Cheng Wang, Wei-Jian Sun, Xiang Zhou, Ming-Dong Lu, Pi-Hong Li, Zhen-Zhai Cai

**Affiliations:** Department of Gastroenterology, The Second Affiliated Hospital & Yuying Children’s Hospital of Wenzhou Medical University, Wenzhou, 325000 Zhejiang Province China; Department of General Surgery, The Second Affiliated Hospital & Yuying Children’s Hospital of Wenzhou Medical University, Wenzhou, 325000 Zhejiang Province China; Departments of Gastroenterology and Hepatology, Jinhua Municipal Central Hospital, Jinhua Hospital of Zhejiang University, Jinhua, 321000 Zhejiang Province China

**Keywords:** Pancreatic cancer, Cancer stem cell, CD133, Bcl-2, Bax, c-myc, Latexin

## Abstract

**Background:**

An increasing number of evidence suggests that pancreatic cancer contains cancer stem cells (CSCs), which may be relevant to the resistance of chemotherapy. Latexin (Lxn) is a negative regulator of stem cell proliferation and we investigate the effects of Lxn on CD133+ pancreatic cancer stem-like cells.

**Methods:**

CD133+ miapaca-2 cells, a human pancreatic carcinoma cell line, were isolated and sorted by magnetic activated cell sorting and flow cytometry. The capacity for self-renewal, proliferation, and tumorigenicity of CD133+ miapaca-2 cells was determined by the floating spheres test and tumor xenograft assays. Protein and mRNA expression of Lxn in CD133+ and CD133- miapaca-2 cells were detected by Western blotting and qRT-PCR, respectively. After CD133+ miapaca-2 cells were treated with Lxn in serum-free medium (SFM), cell proliferation was assayed with a Cell Counting Kit 8 (CCK-8) and apoptosis was analyzed by flow cytometry. The protein and mRNA expression levels of Bcl-2, bax, and c-myc were also analyzed.

**Results:**

We successfully isolated CD133+ miapaca-2 cells that exhibited the capacity for self-renewal in SFM, a proliferation potential in DMEM supplemented with FBS, and high tumorigenicity in nude mice. Lxn protein and mRNA expression levels in CD133+ miapaca-2 cells were significantly lower than those in CD133- cells. Lxn-treated CD133+ miapaca-2 cells exhibited increased apoptosis and low proliferation activity, down-regulation of Bcl-2 and c-myc expression, and up-regulation of Bax expression in a dose-dependent manner.

**Conclusions:**

Lxn induces apoptosis and inhibits the proliferation of CD133+ miapaca-2 cells. These changes are associated with down-regulation of Bcl-2 and c-myc and up-regulation of Bax.

## Background

Pancreatic cancer is a common malignant tumor of the digestive system and the fourth most common cause of cancer death [1]. Due to the lack of early diagnosis, the propensity of early metastatic spread and the high resistance to chemotherapy and radiation therapy, the prognosis of pancreatic cancer remains poor, with the 5-year survival rate being less than 5% [2]. There is increasing evidence supporting a role for cancer stem cells (CSCs) in the initiation, maintenance, proliferation, metastasis, and resistance to therapy of malignant tumors [3]. CSCs have recently been reported in several types of solid tumors, including pancreatic carcinoma [4–8]. Various cell markers have been identified as markers for pancreatic CSCs including CD44, CD24 and the epidermal surface antigen (ESA) [4], CD133 [5], cxc chemokine receptor 4 (CXCR4) [5], c-Met [6], high activity of aldehyde dehydrogenase 1 [7] and low activity of 26S proteasome [8]. Unfortunately, none of the markers described as pancreatic CSC markers are specific to pancreatic adenocarcinoma and there is significant overlap with other cancer types and even with healthy cells. CD133 has been a commonly used marker for CSCs and several studies have reported that CD133+ pancreatic cancer has some characteristics of CSCs [5, 9–11]. There is an increasing interest in searching for regulatory molecules of CSCs and effective approaches for targeting this specific population of cancer cells.

Latexin (Lxn) is an antigen of 29 kDa expressed in a subset of neurons in the rat cerebral cortex as well as in various types of non-neural tissues such as heart, prostate, ovary, kidney, pancreas, and colon [12, 13]. As the only known mammalian carboxypeptidase inhibitor, Lxn shares 30% sequence similarity with tazarotene-induced gene 1 (*TIG1*), which is down-regulated or absent in many classes of tumors [14]. The loss of Lxn expression is associated with an increased incidence of ovarian cancer, leukemia and lymphoma [15]. Lxn expression is reduced in human gastric cancers and hepatocellular carcinoma, compared with normal control tissues [16, 17]. Furthermore, Lxn negatively controls the hematopoietic stem cell (HSC) populations in mice by decreasing cell replication and increasing apoptosis [18, 19]. More recently, Lxn has been shown to inhibit melanoma cell proliferation and down-regulate the expression of several stem cell transcription factors, such as octamer-binding transcription factor 4 (OCT4), sex determining region Y-box 2 (SOX2), Kruppel-like factor 4 (KLF4), NANOG, and MYCN. This evidence indicates that Lxn may indeed alter the stem cell-like properties of melanoma cells [20]. The biological effects of Lxn on pancreatic cancer stem-like cells, however, remain largely unknown.

The Bcl-2 family of proteins plays an important role in the regulation of cell death and is comprised of both pro-apoptotic (such as Bcl-x and Bcl-k) and anti-apoptotic members (such as Bcl-2 and Bcl-xl). Defects in the Bcl-2 family of proteins have been associated with chemotherapy resistance in various human cancers. Reduced expression of anti-apoptotic members promotes apoptotic responses to anticancer drugs, while increased expression of anti-apoptotic members such as Bcl-2 leads to resistance to chemotherapeutic drugs and radiation therapy [21].

Myc has a central function in stem cell biology. For example, the transcription factor c-myc promotes the proliferation and growth of cancer cells. Furthermore, its dysregulation is often associated with advanced malignancy and poor prognosis in diverse human cancers [22]. Civenni *et al*. reported that RNAi-mediated silencing of Myc transcription inhibits stem-like cell maintenance and tumorigenicity in prostate cancer [23]. More interestingly, Bcl-2 activation and overexpression can inhibit apoptosis, prolong cell survival, and reverse the pro-apoptotic effect induced by c-myc [24]. Therefore, we speculated that the Bcl-2 family and c-myc may play a crucial role in the proliferation and apoptosis of CSCs.

The aim of the present study was to explore new strategies for pancreatic cancer therapy. We tested the differential expression of Lxn between CD133+ pancreatic cancer stem-like cells and CD133- pancreatic cancer cells. Furthermore, we investigated whether exogenous Lxn can induce apoptosis and inhibit proliferation in CD133+ pancreatic cancer stem-like cells, and the mechanisms were also studied. It is a meaningful effort to target this specific population of cancer cells for pancreatic cancer therapy.

## Methods

### Cell sorting and cell culture

Human pancreatic cancer cell lines Aspc-1, panc-1, and Bxpc-3 were obtained from the cell bank of the Chinese Academy of Sciences (Shanghai, China). Human pancreatic cancer cell lines miapaca-2 and patu8988 were purchased from the American Type Culture Collection (ATCC; Manassas, VA, USA). All cells were maintained in DMEM supplemented with 10% FBS (Gibco, Grand Island, NY, USA), 100 units/mL of penicillin G and 10 μg/mL of streptomycin (Gibco, Grand Island, NY, USA). The contents of CD133+ cells in the 5 human pancreatic cancer cell lines were measured by flow cytometry (FCM). CD133+ cells of the human pancreatic carcinoma cell line miapaca-2 were isolated by magnetic activated cell sorting (MACS). A total of 5 × 10^7^ miapaca-2 cells were trypsinized, washed, and resuspended in 300 μL of PBS. After an addition of 100 μL of FcR blocking reagent, the cells were incubated in 100 μL of monoclonal CD133 antibodies labeled with MicroBeads (Miltenyi Biotec Ltd, Bergisch Gladbach, Germany: CD133/1; clone AC133, order number 130-050-801) for 30 minutes at 4°C. The CD133+ cells were enriched using a MiniMACS magnet and MS columns (Miltenyi Biotech Ltd, Bergisch Gladbach, Germany) and were flushed out by applying the plunger supplied with the column. The purity of CD133+ cells isolated from miapaca-2 cells was determined by FCM analysis using a phycoerythrin (PE)-labeled antibody against human CD133/2 (clone 293C3; Miltenyi Biotec Ltd, Bergisch Gladbach, Germany, CD133/2(293C3)-PE, order number 130-090-853). A PE-labeled antibody against mouse IgG2b was used as an isotype. All procedures were performed according to the manufacturers’ instructions. The FCM analysis was performed using BD FACSAria (Becton, Dickinson and Company, Franklin Lakes, NJ, USA). The data were analyzed by Flowjo7.6.5 software (Tree Star Inc., Ashland, OR, USA).

CD133+ and/or CD133- cells isolated by MACS were seeded at a density of 1,000 cells/mL and cultured in serum-free medium (SFM) containing DMEM/F12 (Gibco, Grand Island, NY, USA), 20 ng/mL of epidermal growth factor (EGF; PeproTech Asia, Rehovot, Israel), 1% of B27 (Gibco, Grand Island, NY, USA), 0.4% of FBS (Sigma-Aldrich, St. Louis, MO, USA), 5 μg/mL of bovine insulin (Sigma-Aldrich, St. Louis, MO, USA, USA), and 100 U/mL of penicillin-streptomycin at 37°C in a humidified atmosphere containing 5% CO_2._

### Tumor xenograft experiment

All experimental procedures and protocols involving animals were reviewed and approved by the Ethics Committee of Wenzhou Medical University, Wenzhou, China (number WYDW-20120008). Male athymic BALB/c nu/nu mice (4 to 6 weeks old) were purchased from the Shanghai Laboratory Animal Co. Ltd. (SLAC) (Shanghai, China). CD133+, CD133- and unsorted miapaca-2 cells were injected subcutaneously into the right anterior flank of mice in various cell number totals (1 × 10^4^, 1 × 10^5^, and 1 × 10^6^ in 150 μL PBS, respectively). The number of mice exhibiting tumors was documented at 30 days after cell injection. The animal protocol was designed to minimize pain or discomfort to the animals. The animals were acclimatized to laboratory conditions (23°C, 12 hours/12 hours light/dark, 50% humidity, *ad libitum* access to food and water) for 2 weeks prior to experimentation. All animals were euthanized by barbiturate overdose (intravenous injection, 150 mg/kg pentobarbital sodium) for tissue collection.

### Detection of apoptosis

CD133+ miapaca-2 cells in logarithmic phase were incubated with different concentrations of Lxn (Abcam, London, UK, ab87145; 0 ng/μL, 5 ng/μL, 10 ng/μL, 20 ng/μL and 40 ng/μL) in SFM for 48 hours; cells of treated and control groups were collected and digested with ethylene diamine tetraacetic acid (EDTA) trypsinase. Cells were collected, washed twice with ice-cold PBS, and then precipitated by centrifugation at 500 g for 10 minutes; the cell pellets were resuspended in 1 × Annexin V binding buffer. To a 100-μL aliquot of the cell suspension, 5 μL of Annexin V (20 μg/mL; Beyotime, Jiangsu, China) and 5 μL of propidium iodide (PI; 50 μg/mL; Beyotime, Jiangsu, China) were added, and the cells were incubated in the dark for 15 minutes at room temperature (25°C). Flow cytometry was performed using FACSCalibur (Becton-Dickinson, San Jose, CA, USA). The data from a total of 10,000 events were analyzed using CellQuest software (Becton-Dickinson Immunocytometry Systems, San Jose, CA, USA). The percentage of Annexin V-positive or PI-positive cells was calculated.

### Cell proliferation assay

A Cell Counting Kit 8 (CCK-8; Dojindo, Kumamoto, Japan) was used to assay the antiproliferative activity of Lxn. The cells were plated at a density of 5,000 cells per well in 96-well plates containing SFM. Then, different concentrations of Lxn (0 ng/μL, 5 ng/μL, 10 ng/μL, 20 ng/μL, and 40 ng/μL) were added to the wells to a final volume of 100 μL per well and incubated for 24 hours, 48 hours, and 72 hours. Subsequently, 10 μL of CCK-8 reagent were added to each well and incubated for 4 hours. The optical density (OD) at a wavelength of 450 nm was recorded using a microplate reader (ELX800; Bio-Tek, Shoreline, WA, USA). All experiments were performed in triplicate on three independent experiments.

### qRT-PCR

Total RNA was isolated with TRIzol (Invitrogen, Carlsbad, CA, USA) according to manufacturer's instructions. A reverse transcription reaction was performed with a ertAid™ First Strand cDNA Synthesis Kit from Fermentas (Burlington, ON, Canada) using 2 μg of total RNA in a final reaction volume of 20 μL. qRT-PCR was performed with SYBR® Premix Ex Taq™ (perfect real time) PCR kit from TaKaRa (Dalian, China) and the LightCycler 480 (Roche Biochemicals, Indianapolis, IN, USA). The primer sequences were as follows: Lxn, sense 5’-GAAGGTCAAACAAGCCAGCA-3’, antisense 5’-AACCCAGGCTAAATGTAGAACG-3’; CD133, sense 5’-CACTTACGGCACTCTTCACCTG-3’, antisense 5’-TGAAGTATCTTGACGCTTTGGTAT-3’; Bcl-2, sense 5’-CGCAGAGGGGCTACGAGT-3’, antisense 5’-GTTGACGCTCTCCACACACAT-3’; bax, sense 5’-TTTCTGACGGCAACTTCAACTG-3’, antisense 5’-CAACCACCCTGGTCTTGGAT-3’; c-myc, sense 5’-GGTCTTCCCCTACCCTCTCA-3’, antisense 5’-CTCCAGCAGAAGGTGATCCA-3’; and β-actin, sense 5’-CGTGGACATCCGCAAAGAC-3’, antisense 5’-AAGAAAGGGTGTAACGCAACTAAG-3’. The qRT-PCR conditions were 30 seconds at 95°C, followed by 45 cycles of 95°C for 10 seconds, and 60°C for 20 seconds. The melting curve analysis was performed to verify product purity and exclude undesired primer dimers. All analyses were performed in triplicate in three independent experiments. The relative amount of target gene mRNA was normalized to that of controls (β-actin).

### Western blotting analysis

Harvested cells were washed with cold PBS and then lysed with radioimmunoprecipitation assay (RIPA) Lysis Buffer (Beyotime Bio, Haimen, China) containing 50 mM Tris (pH 7.4), 15 mM NaCl, 1% Triton X-100, 1% sodium deoxycholate, 0.1% sodium dodecyl sulfate (SDS) and protease inhibitors. The cell lysates were centrifuged at 12,000 g at 4°C for 20 minutes and the total proteins of the supernatants were measured with a Beyotime BCA Protein Assay Kit (Beyotime Bio, Haimen, China), according to the manufacturer’s protocol. Equal amounts (30 μg) of protein were electrophoresed on a 10% SDS-PAGE gel and transferred to a polyvinylidene fluoride (PVDF) membrane (Beyotime Bio, Haimen, China), which was incubated overnight at 4°C with the following primary antibodies: anti-Lxn antibody (Abcam, London, UK, ab103485, diluted 1:500), anti-Bcl-2 antibody (Abcam, London, UK, ab18210, diluted 1:1,000), anti-bax antibody (Abcam, ab7977, diluted 1:1,000), anti-c-Myc (9E10) antibody (Abcam, ab32, diluted 1:1,000), and anti-β-actin antibody (Beyotime, Haimen, China, aa128, diluted 1:1,000). Subsequently, the membrane was incubated at room temperature for 2 hours with the following horseradish peroxidase (HRP) labeled secondary antibodies: HRP-labeled Goat Anti-Mouse IgG (Beyotime Bio, Haimen, China, a0216, diluted 1:1,000) and HRP-labeled Goat Anti-Rabbit IgG (Beyotime Bio, Haimen, China, aa208, diluted 1:1,000). The protein bands were detected by enhanced chemiluminescence detection reagents (Applygen Technologies, Beijing, China) and documented with AlphaEaseFC 4.0 software (Alpha Innotech Co., San Leandro, CA, USA).

### Statistical analysis

Data are expressed as the mean ± SD. SPSS 16.0 for Windows (SPSS Inc., Chicago, IL, USA) was used for statistical analysis. Differences between treated groups were analyzed with a one-way analysis of variance (ANOVA). Fisher's exact probability was used to compare the differences in categorized data. *P* < 0.05 was considered statistically significant.

## Results

### Separation of CD133+ miapaca-2 and CD133- cells by MACS and evaluation of the efficiency of sorting by flow cytometry

**Figure 1:**
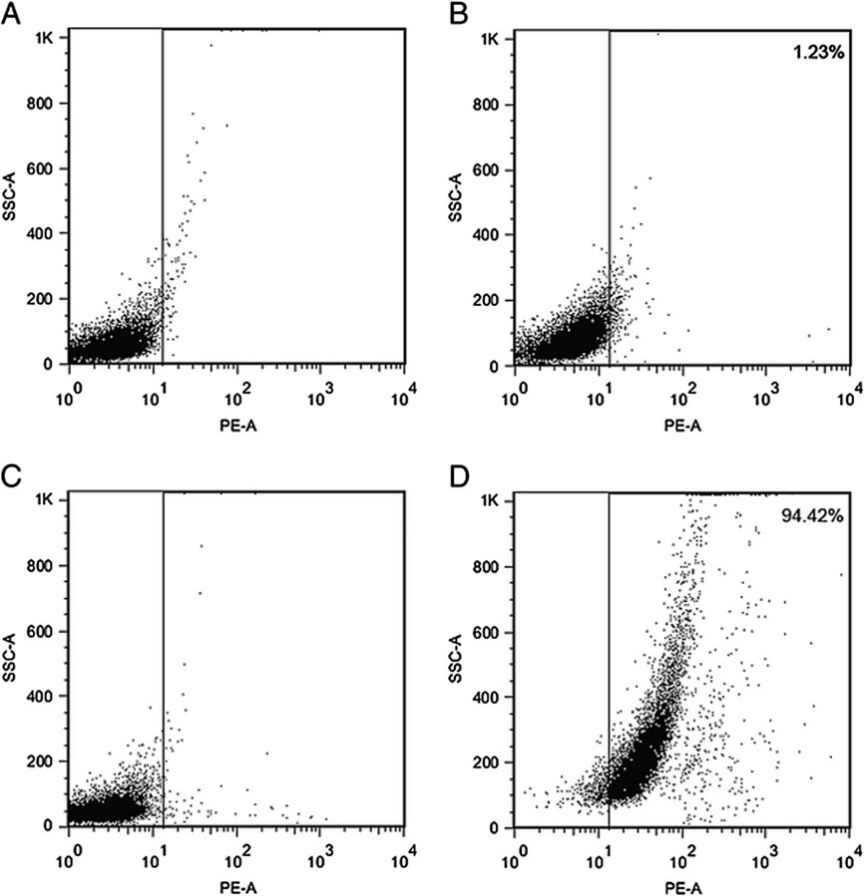
**The percentage of CD133+ cells in miapaca-2 and sorted CD133+ cells determined by flow cytometry using CD133/2 (293C3)-PE antibodies.** The mouse IgG2b-PE antibody was used as an isotype. **(A)** Miapaca-2 mouse IgG2b-PE (isotype), **(B)** Miapaca-2 CD133-PE, **(C)** Miapaca-2 CD133+ cells mouse IgG2b-PE (isotype), **(D)** Miapaca-2 CD133+ cells CD133-PE.

The percentages of CD133+ cells in miapaca-2, aspc-1, panc-1 cells, bxpc-3 and patu8988 cells were 1.18 ± 0.23%, 1.75 ± 0.45%, 1.24 ± 0.31%, 0.25 ± 0.13% and 0.29 ± 0.15%, respectively. To obtain CD133+ pancreatic cancer cells, a large number of cells are needed for MACS because of the low proportion of CD133+ pancreatic cancer cells. Miapaca-2 cells are easier to cultivate and have a shorter cell growth cycle. We therefore chose miapaca-2 cells for the following experiments. CD133+ and CD133- miapaca-2 cells were separated by MACS and the purity of sorted CD133+ miapaca-2 cells was found to be 94.45 ± 0.35% (Figure 1).

### CD133+ cells in serum-free medium: formation of floating spheres

**Figure 2:**
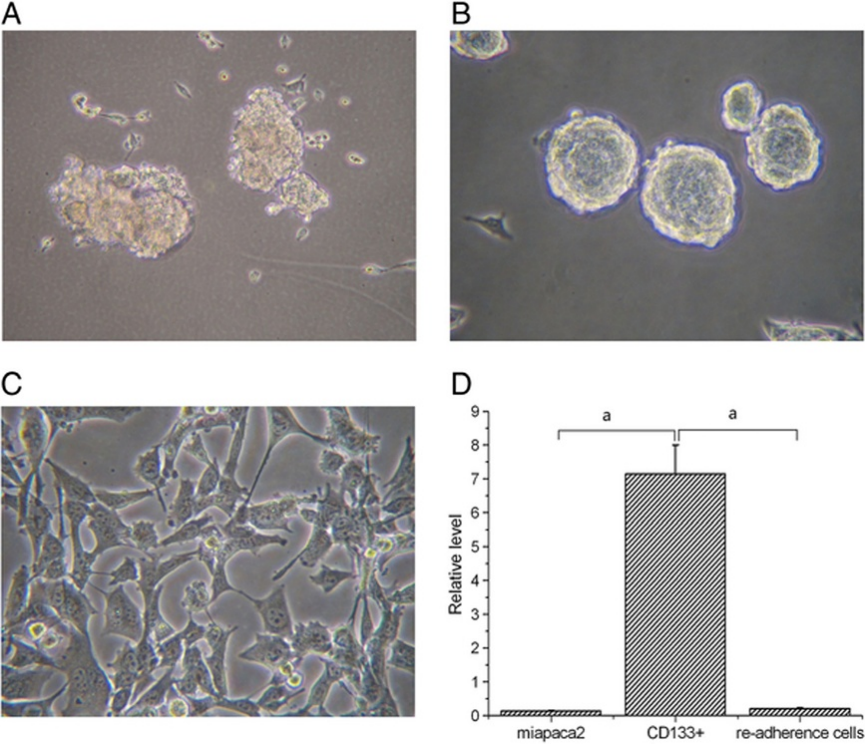
**Proliferation ability of the CD133+ and CD133- cells in serum-free medium.** CD133- cells showed slow growth and good adherence; and almost all cells died within 2 weeks **(A)**. CD133+ cells formed floating spheres **(B)**. When the floating spheres were cultured in DMEM supplemented with 10% FBS, the cells were adherent and proliferated similarly to unsorted miapaca-2 cells **(C)**. The expression of CD133 mRNA in CD133+ cells was dramatically higher than unsorted miapaca-2 cells and re-adherence CD133+ cells **(D)**. ^a^ *P* < 0.05.

We compared the proliferative capacity of CD133+ and CD133- cells in SFM. Cells were cultured at a density of 1,000 cells/mL in 96-well plates for 7 days. As shown in Figures 2A and B, the two populations of cells exhibited different morphologies. CD133- cells exhibited markedly slower proliferation, greater adherence, and a higher rate of cell death after 1 week in culture. Under the same culture conditions, the CD133+ cells formed floating spheres (Figure 2B). After the floating spheres were harvested and gently disaggregated to a single cell suspension, the cells were cultured in DMEM supplemented with 10% FBS 100 units/mL penicillin G and 10 μg/mL of streptomycin. The cells were adherent and proliferated without significant differences from the unsorted miapaca-2 cells (Figure 2C). Furthermore, the relative level of CD133 mRNA in CD133+ cells was 7.15 ± 0.85,which was dramatically higher than that in unsorted miapaca-2 cells (0.14 ± 0.02; *P* = 0.000) and re-adherence cells from the floating spheres (0.21 ± 0.03; *P* = 0.001; Figure 2D).

### Tumorigenicity of CD133+ cells in athymic BALB/c nu/nu mice

**Figure 3:**
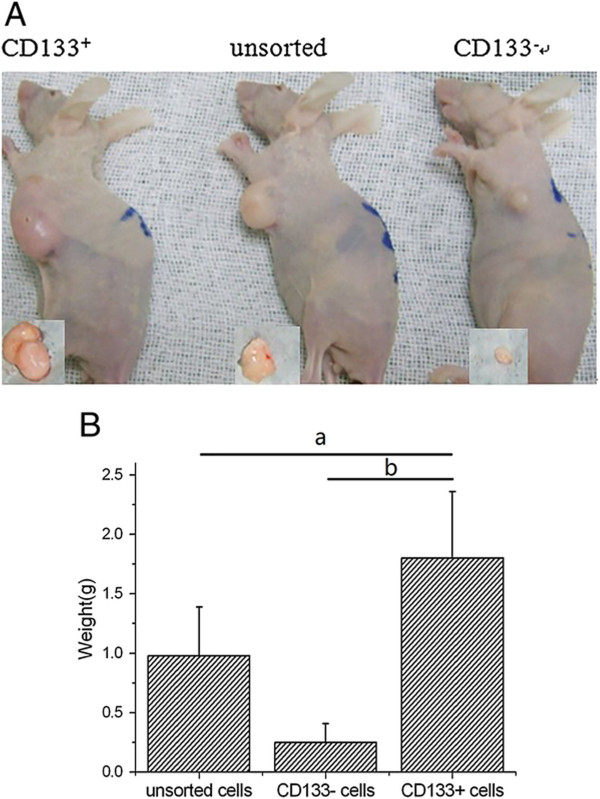
**Differences of tumor size and tumor weight between CD133 + 、** **CD133- and unsorted miapaca-2 cell groups. (A)** Tumor size of CD133+ cell groups were greater than that of CD133- cell groups and unsorted miapaca-2 cell groups by naked eye. **(B)** Tumor weight of CD133+ cell groups were more than that of CD133- cell groups and unsorted miapaca-2 cell groups. ^a^ *P* < 0.05, ^b^ *P* < 0.05.

Nude mice were injected subcutaneously with CD133+ cells, CD133- cells or unsorted miapaca-2 cells at concentrations of 1 × 10^4^, 1 × 10^5^, or 1× 10^6^, respectively (in 150 μL PBS). Tumorigenicity, as measured at 30 days after cell injection, was significantly different among the three types of cells: 1 × 10^4^ CD133+ cells formed tumors, 1 × 10^6^ CD133- cells and 1 × 10^5^ unsorted miapaca-2 cells formed tumors in some mice, while 1 × 10^4^ CD133- cells or unsorted miapaca-2 cells failed to form tumors (Table 1). The tumor weight (1.8 ± 0.52 g) of CD133+ cell groups was more than that of CD133- cell groups (0.25 ± 0.16 g, *P* = 0.000) and unsorted miapaca-2 cell groups (0.98 ± 0.41 g, *P* = 0.008; Figure 3). These results indicated that CD133+ cells were associated with high tumorigenicity.

**Table 1:** **Tumorigenic ability of CD133+ cells, CD133- cells and unsorted miapaca-2 cells**

CD133+ cells	CD133- cells	Unsorted miapaca-2 cells
1 × 10^4^ cells 9/10	0/10^a^	0/10^b^
1 × 10^5^ cells 10/10	0/10^a^	4/10^b^
1 × 10^6^ cells 10/10	4/10^a^	10/10

^a^ *P* < 0.05, ^b^ *P* < 0.05 versus CD133+ cells.

### The expression of Lxn in CD133+ and CD133- cells

**Figure 4:**
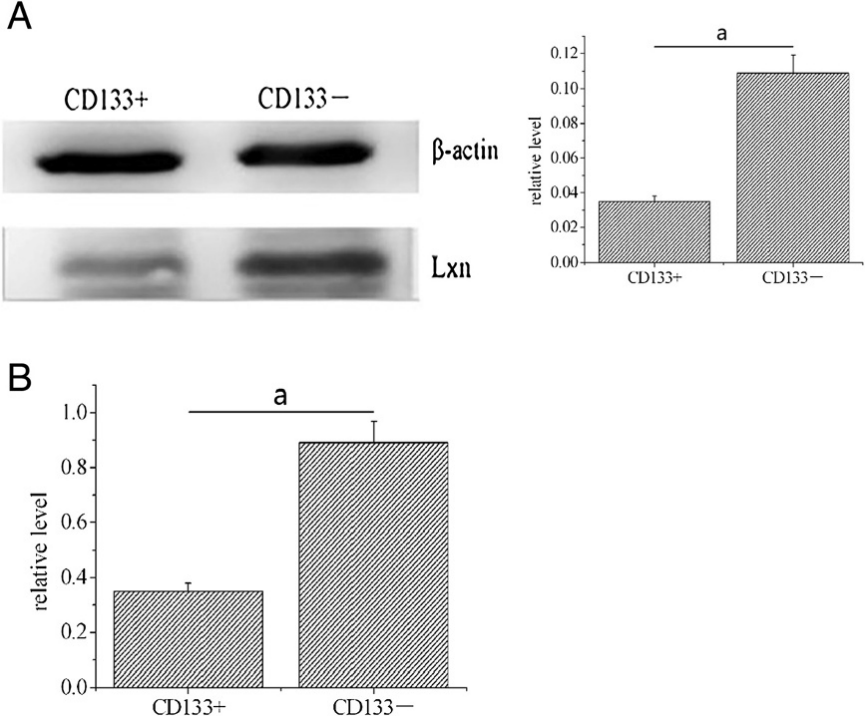
**Lxn expression in CD133+ and CD133- cells. (A)** The Lxn protein expression level was decreased in CD133+ cells compared to CD133- cells. **(B)** The Lxn mRNA expression level in CD133+ cells was significantly lower than that in CD133- cells. ^a^ *P* < 0.05.

The relative level of Lxn protein in CD133+ cells was significantly lower than that in CD133- cells (0.35 ± 0.03 versus 0.89 ± 0.08; *P* < 0.05; Figure 4A, B). Similarly, the relative level of Lxn mRNA in CD133+ cells was significantly lower than that in CD133- cells (0.035 ± 0.003 versus 0.109 ± 0.012; *P* < 0.05; Figure 4C).

### Effects of Lxn on apoptosis and cell growth in CD133+ cells

**Figure 5:**
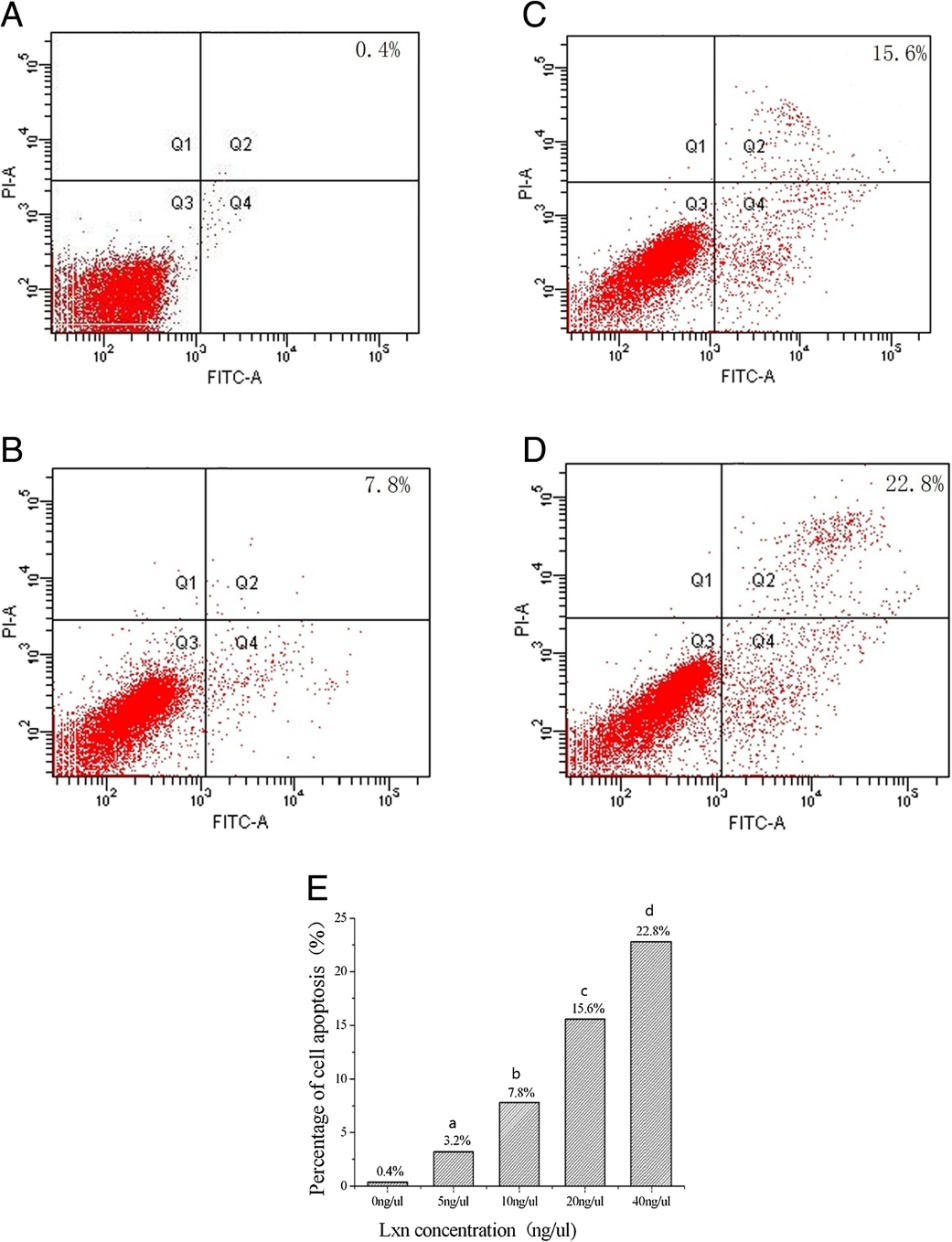
**Cell death analysis in CD133+ and CD133- cells. (A-D)** Apoptosis of CD133+ miapaca-2 cells treated with 0 ng/μL **(A)**, 10 ng/μL **(B)**, 20 ng/μL **(C)** and 40 ng/μL **(D)** of Lxn. The lower left indicates live cells (Annexin V-FITC negative/PI negative); the lower right shows early apoptotic cells (Annexin V-FITC positive/PI negative). The upper left shows damaged cells (Annexin V-FITC negative/PI positive), while the upper right demonstrates necrotic cells and late apoptotic cells (Annexin V-FITC positive/PI positive). The number represents the percentage of early apoptotic cells, necrotic cells, and late apoptotic cells in each condition (right quadrant). **(E)** The percentage of apoptotic cells, necrotic cells, and late apoptotic CD133+ miapaca-2 cells treated with 0 ng/μL, 5 ng/μL, 10 ng/μL, 20 ng/μL and 40 ng/μL of Lxn. ^a^ *P* < 0.05, ^b^ *P* < 0.05, ^c^ *P* < 0.05, ^d^ *P* < 0.05. FITC, fluorescein iodothiocyanate; PI, propidium bromide.

We determined the effects of Lxn on the rate of apoptosis in CD133+ cells relative to controls. As shown in Figures 5B, C and D, the treatment of cells with Lxn for 48 hours increased the percentage of early and late apoptotic cells as well as necrotic cells in a dose-dependent manner. On average, 22.8% of cells treated with 40 ng/μL of Lxn were classified as necrotic cells or late apoptotic cells.

**Figure 6:**
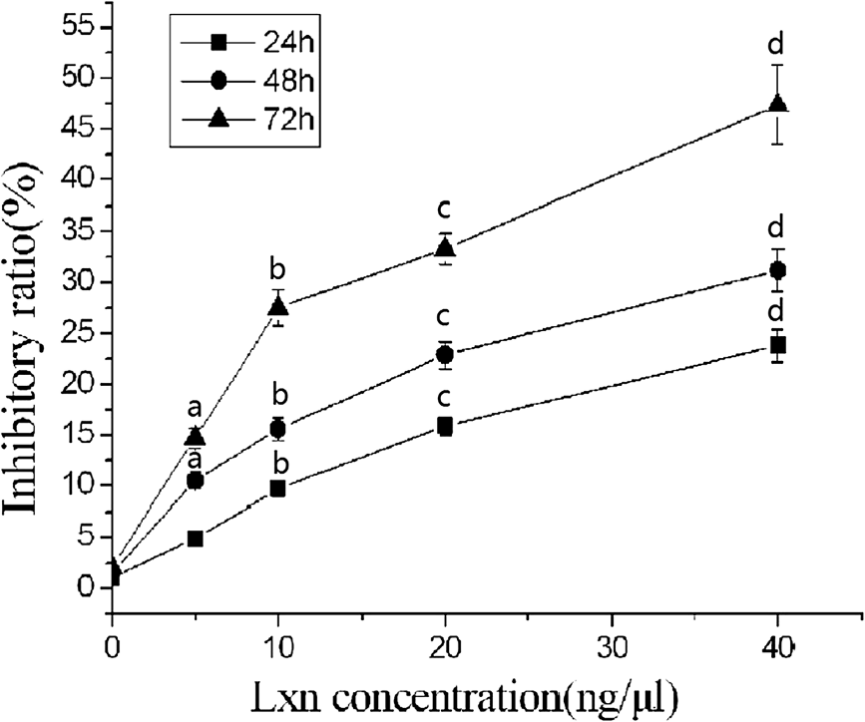
**Cytotoxic effects of Lxn treatment in CD133+ miapaca-2 cells treated with Lxn at various concentrations of Lxn.** The inhibitory ratio was calculated according to the following formula: Inhibitory ratio = (OD450 nm value of control group - OD450 nm value of Lxn treatment group)/(OD450 nm value of control group - OD450 nm value of blank group). ^a^ *P* < 0.05, ^b^ *P* < 0.05, ^c^ *P* < 0.05, ^d^ *P* < 0.05.

We also determined the cell killing effects of Lxn on CD133+ cells. As shown in Figure 6, with increasing Lxn concentrations, its inhibitory effects on CD133+ cells increased; the treatment with 5 ng/μL and 40 ng/μL of Lxn for 48 hours resulted in a cell growth inhibition at a rate of 10.5% and 31.2%, respectively. Similarly, the inhibitory effects on CD133+ cells increased as a function of the incubation period. For example, treatment with 40 ng/μL of Lxn for 24 hours and 72 hours resulted in cell inhibition rates of 23.8% and 47.4%, respectively. Therefore, Lxn suppressed the proliferation of CD133+ cells in a time- and concentration-dependent manner.

### Effects of Lxn on the expression of Bcl-2, bax, and c-myc in CD133+ cells

**Figure 7:**
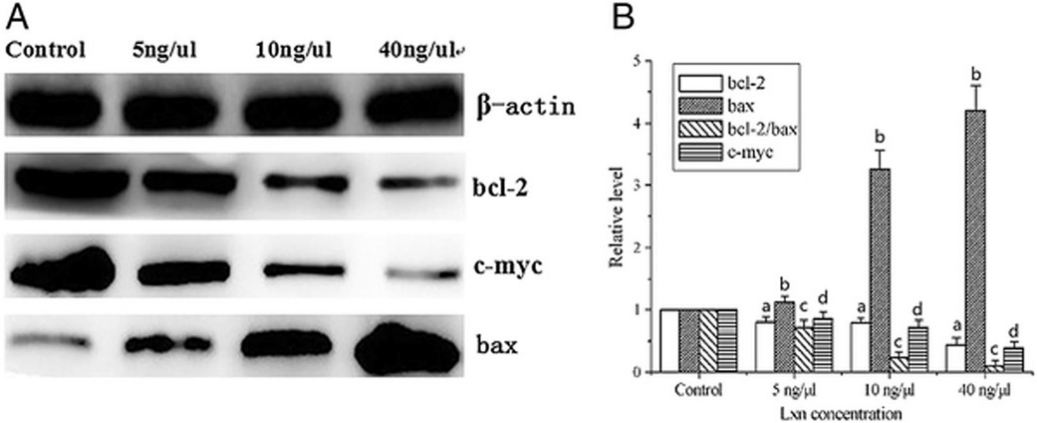
**The expression level of Bcl-2、c-myc and bax protein after treatment with Lxn in CD133+ cells. (A)** The expression level of Bcl-2、c-myc and bax protein by Western blot analysis. **(B)** The relative expression level of Bcl-2、bax、bcl-2/bax and c-myc protein compared to the controls. ^a^ *P* < 0.05, ^b^ *P* < 0.05, ^c^ *P* < 0.05, ^d^ *P* < 0.05.

**Figure 8:**
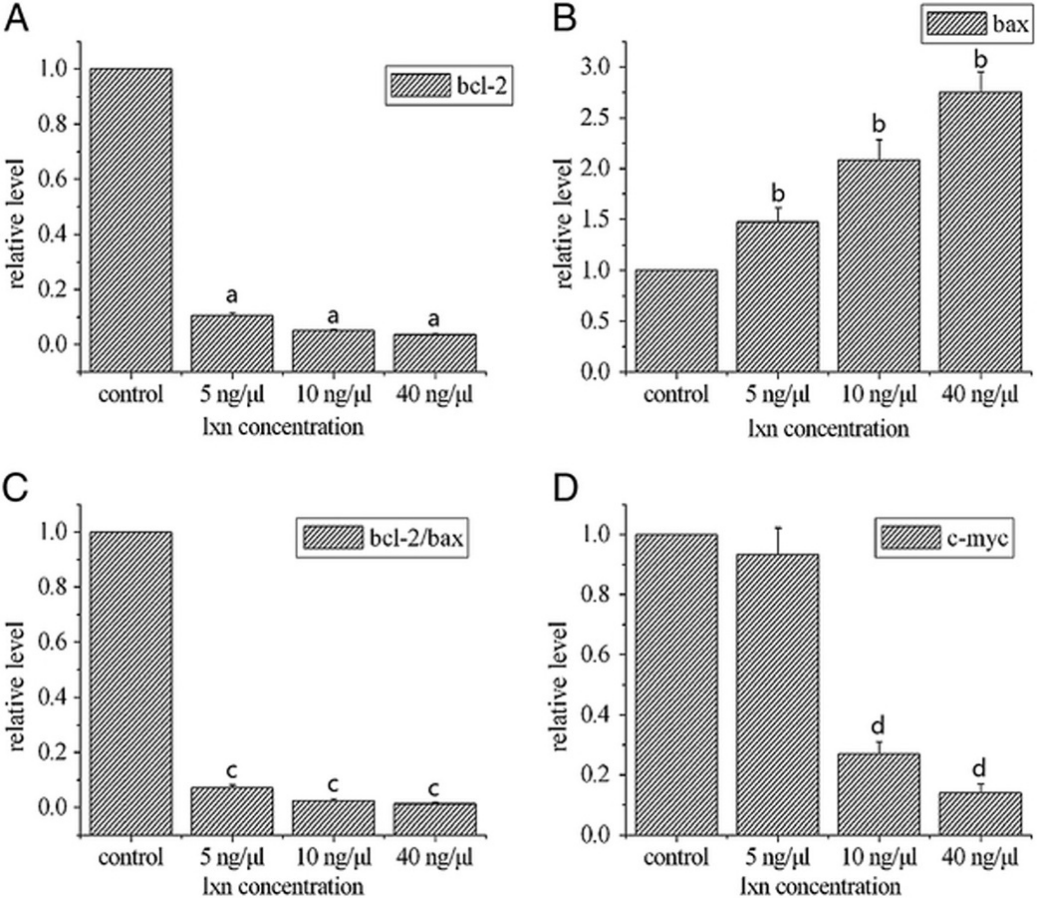
**The mRNA expression of Bcl-2 (A) and bax (B).** The ratio of Bcl-2/bax **(C)** and c-myc **(D)**. The relative amount of target gene mRNA was normalized to β-actin and compared with the controls. ^a^ *P* < 0.05, ^b^ *P* < 0.05, ^c^ *P* < 0.05, ^d^ *P* < 0.05.

As shown in Figure 7, Western blot analysis revealed concentration-dependent decreases in Bcl-2 protein, Bcl-2/bax ratio, and c-myc protein and an increase in bax protein in CD133+ cells after treatment with Lxn. Even a low concentration of Lxn (5 ng/μL) resulted in the inhibition of Bcl-2 (0.81 ± 0.11 versus control, *P* = 0.021) and c-myc protein expression (0.85 ± 0.12 versus control, *P* = 0.038). Moreover, treatment of cells with 40 ng/μL Lxn resulted in markedly greater reduction in the expression of both proteins (0.44 ± 0.06 versus control (*P* = 0.000) and 0.39 ± 0.05 versus control (*P* = 0.000), respectively). Meanwhile, the expression of bax protein was significantly increased in the 10 ng/μL (3.28 ± 0.3, *P* = 0.000) and 40 ng/μL (4.2 ± 0.4, *P* = 0.000) groups compared with that of controls, and the Bcl-2/bax ratio was decreased. Analogous to changes in protein expression, qRT-PCR analyses also showed a concentration-dependent decrease in the expression of Bcl-2 (versus control, *P* < 0.05), Bcl-2/bax ratio (versus control, *P* < 0.05), and c-myc mRNA (versus control, *P* < 0.05) and an increase in the expression of bax mRNA levels (versus control, *P* < 0.05) (Figure 8).

## Discussion

CSCs are characterized by extensive self-renewal and proliferative capacity as well as resistance to chemotherapy and radiotherapy. Indeed, CSCs play an important role in the initiation, maintenance and relapse of cancers [25]. The inability to effectively target CSCs is thought to contribute to the failure of existing therapies [26]. In the present study, we successfully isolated CD133+ miapaca-2 cells and demonstrated that they had some characteristics of CSCs. Using these cancer stem-like cells, we further demonstrated the effects of Lxn on the growth of these cells and apoptosis, which were associated with the Bcl-2 family and c-myc. We believe that these results may be useful in exploring novel therapeutic strategies for pancreatic cancer by targeting CSCs.

In the present study, we found that Lxn was decreased in CD133+ cells compared to CD133- cells, suggesting latexin may play a role in the suppression of pancreatic cancer stem-like cells. To validate this point, CD133+ cells were treated with different concentrations of Lxn protein. Our data indicated that Lxn increased cell death. Lxn was originally identified in the lateral neocortex of rats and serves as a marker of rationality and development in rodent nervous systems. It acts as a tissue or endogenous carboxypeptidase inhibitor in humans [27]. It is reported that latexin negatively controls the HSC populations in mice by decreasing cell replication and increasing apoptosis [18]. Furthermore, the ectopically expressed latexin in mouse lymphoma cells which are lacking native latexin expression results in remarkable suppression of cell growth [28]. The Lxn expression is reduced in human gastric cancers compared with their normal control tissues [16]. Lxn also inhibits gastric cancer cell growth and tumorigenicity [16].

The Bcl-2 family plays a central role in the regulation of cell death pathways including apoptosis, necrosis and autophagy [29]. One of the major cell-death pathways, namely the mitochondrial apoptotic pathway, is initiated by mitochondrial outer-membrane permeabilization. This, in turn, allows soluble proteins such as cytochrome C in the mitochondrial inter-membrane space to diffuse into the cytosol; thereby, engaging apoptotic protease activating factor-1 (APAF-1) to oligomerize into a caspase activation platform termed apoptosome. Apoptosome binds and promotes the activation of initiator caspase-9 and triggers a cascade of caspase (which are caspase-3, caspase-6, and caspase-7) activation, resulting in the morphologic and biochemical changes associated with apoptosis [30]. The overexpression of anti-apoptotic Bcl-2 family members has been associated with chemotherapy resistance in various human cancers. Indeed, targeting the anti-apoptotic Bcl-2 family members can improve apoptosis and, thus, overcome drug resistance to cancer chemotherapy [21]. The balance between anti-apoptotic and pro-apoptotic Bcl-2 family members, rather than mere overexpression of Bcl-2, regulates cancer cell death [29]. In the present study, we evaluated changes in the expression of Bcl-2 and bax after treatment of CD133+ cells with Lxn. We demonstrated a concentration-dependent decrease in the expression of Bcl-2 at the protein and mRNA levels and an increase in the expression of bax, thereby resulting in a decrease in the ratio of Bcl-2/bax. Our findings suggested that Lxn regulates apoptosis and the proliferation of CD133+ miapaca-2 cells in a manner that is dependent on the Bcl-2 family of proteins. Future studies should examine the possible mechanisms of action.

In the present study, we evaluated changes in c-myc following treatment of CD133+ cells with Lxn, and demonstrated a concentration-dependent decrease in c-myc expression. These results indicated that Lxn regulates CD133+ miapaca-2 cell apoptosis and proliferation via c-myc. The proto-oncogene c-myc has been recognized as an important regulator of stem cells, serving as a link between malignancy and 'stemness' [31]. The activity of c-myc is required for proliferation, growth, and survival of glioma cancer stem cells [32]. Future studies should further examine the mechanisms responsible for the link between Lxn, the Bcl-2 family and c-myc in CSCs.

## Conclusions

The present study indicated that Lxn induces apoptosis and inhibits the proliferation of CD133+ miapaca-2 pancreatic cancer stem-like cells' correlation to the Bcl-2 family and c-myc. Targeting Lxn may represent a new therapeutic strategy for pancreatic cancer.

## Abbreviations

ANOVA: analysis of variance
CCK-8: Cell Counting Kit 8
CSCs: cancer stem cells
CXCR4: cxc chemokine receptor 4
DMEM: Dulbecco's modified Eagle medium
EDTA: ethylene diamine tetraacetic acid
ESA: epidermal surface antigen
FBS: fetal bovine serum
FCM: flow cytometry
HRP: horseradish peroxidase
HSC: hematopoietic stem cell
KLF4: Kruppel-like factor 4
SFM: serum-free medium
Lxn: latexin
MACS: magnetic activated cell sorting
OCT4: octamer-binding transcription factor 4
OD: optical density
PBS: phosphate-buffered saline
PCR: polymerase chain reaction
PE: phycoerythrin
PI: propidium iodide
PVDF: polyvinylidene ifluoride
RIPA: radioimmunoprecpitation assay
SDS: sodium dodecyl sulfate
SOX2: sex determining region Y-box 2
*TIG1*: tazarotene-induced gene 1.
